# Alzheimer’s Disease and *Porphyromonas gingivalis*: Exploring the Links

**DOI:** 10.3390/life15010096

**Published:** 2025-01-14

**Authors:** Ivana Shawkatova, Vladimira Durmanova, Juraj Javor

**Affiliations:** Institute of Immunology, Faculty of Medicine, Comenius University in Bratislava, Odborarske nam. 14, 811 08 Bratislava, Slovakia; vladimira.durmanova@fmed.uniba.sk (V.D.); juraj.javor@fmed.uniba.sk (J.J.)

**Keywords:** Alzheimer’s disease, gingipains, oral pathogen, pathogenesis, periodontitis, *Porphyromonas gingivalis*, virulence factors

## Abstract

Recent research highlights compelling links between oral health, particularly periodontitis, and systemic diseases, including Alzheimer’s disease (AD). Although the biological mechanisms underlying these associations remain unclear, the role of periodontal pathogens, particularly *Porphyromonas gingivalis*, has garnered significant attention. *P. gingivalis*, a major driver of periodontitis, is recognized for its potential systemic effects and its putative role in AD pathogenesis. This review examines evidence connecting *P. gingivalis* to hallmark AD features, such as amyloid β accumulation, tau hyperphosphorylation, neuroinflammation, and other neuropathological features consistent with AD. Virulence factors, such as gingipains and lipopolysaccharides, were shown to be implicated in blood–brain barrier disruption, neuroinflammation, and neuronal damage. *P. gingivalis*-derived outer membrane vesicles may serve to disseminate virulence factors to brain tissues. Indirect mechanisms, including systemic inflammation triggered by chronic periodontal infections, are also supposed to exacerbate neurodegenerative processes. While the exact pathways remain uncertain, studies detecting *P. gingivalis* virulence factors and its other components in AD-affected brains support their possible role in disease pathogenesis. This review underscores the need for further investigation into *P. gingivalis*-mediated mechanisms and their interplay with host responses. Understanding these interactions could provide critical insights into novel strategies for reducing AD risk through periodontal disease management.

## 1. Introduction

In recent years, mounting evidence has connected oral diseases with a spectrum of seemingly unrelated systemic conditions, including cardiovascular diseases, type 2 diabetes, malignancies, rheumatoid arthritis, and adverse pregnancy outcomes [1,2,3,4,5,6,7,8]. This connection is particularly evident in periodontitis, a chronic inflammatory disease that has also been linked to an increased risk of brain amyloid β (Aβ) accumulation, cognitive impairment, all-cause dementia, and Alzheimer’s disease (AD) [9,10,11,12,13,14,15,16,17,18,19]. While the precise biological mechanisms underlying these associations remain incompletely understood, current research highlights the role of periodontal pathogens. Among these, *Porphyromonas gingivalis*, an opportunistic pathogen and major driver of periodontitis, has garnered attention for its potential impact on systemic health and its putative role in the pathogenesis of AD [20].

The last decade has witnessed significant advancements in the understanding of the potential involvement of *P. gingivalis* in AD pathogenesis and the development of disease-related hallmark pathologies. The experimental oral infection of mice with *P. gingivalis* has demonstrated brain colonization, increased extracellular Aβ accumulation, and other AD-like phenotypes, suggesting that this bacterium may disseminate from established peripheral infection sites into the CNS and initiate neuropathological changes consistent with those observed in AD in humans [21,22,23,24,25]. In support of this, *P. gingivalis*, its genomic DNA, and key virulence factors have also been detected in the post-mortem brain tissue of patients with AD. [25,26,27]. Furthermore, in vitro studies have shown that *P. gingivalis* can invade and persist within neurons, potentially leading to neurodegeneration [28].

This review aims to explore the current evidence supporting a link between *P. gingivalis* periodontal infection and Alzheimer’s disease. It focuses on the bacterium’s key virulence factors and their possible roles in the molecular mechanisms contributing to the development and progression of AD pathology. Understanding these interactions may provide insights into novel intervention strategies aimed at mitigating AD risk through the management of periodontal disease.

## 2. Alzheimer’s Disease

Alzheimer’s disease is the most prevalent neurodegenerative disorder worldwide, accounting for an estimated 60% to 80% of dementia cases [29]. It is commonly understood that AD predominantly affects elderly people. According to current data, approximately 10% of individuals aged 65 and older, and nearly 35% of those aged 85 and above, are affected by the disease [29,30]. Due to the progressive aging of the global population, the number of people affected by AD will supposedly rise significantly in the coming decades, mainly in developed countries [31].

AD is histologically characterized by the presence of pathologic protein aggregates in the brain. These include the extracellular accumulations of Aβ, known as amyloid plaques, and intracellular hyperphosphorylated tau constituting neurofibrillary tangles (NFTs) within neurons [32]. Protein aggregates are commonly accompanied by dystrophic neurites, microglia activation, and associated astrogliosis. In simple terms, the presence of toxic beta-amyloid and tau causes the activation of microglia, which strive to clear the proteins and debris from dead cells. However, chronic inflammation may follow when microglia are overwhelmed and cannot keep up with clearance demands [29]. Both animal models and clinical evidence show that alongside the typical neuropathological hallmarks, a characteristic feature of AD is neuroinflammation and neuronal death. A reduction in the number of neurons and synapses is particularly significant in the hippocampus and cortex. Since the hippocampus is essential for learning and memory, AD clinically manifests as a progressive decline in memory and cognitive skills [33]. Eventually, neuronal damage extends to the regions of the brain responsible for essential physiological processes. Brain function is further impaired by reduced glucose metabolism, which is critical for sustaining neural activity [34]. Ultimately, neurodegeneration and other factors result in accelerated brain atrophy—another pathological feature associated with advanced AD.

Based on the age at disease onset and type of heritability, AD is commonly categorized into two forms: familial early-onset (EOAD) and sporadic late-onset Alzheimer’s disease (LOAD). The vast majority of AD cases (more than 95%) are represented by the late-onset form in which symptoms become apparent when patients are in their mid-60s and later. Most EOAD cases are due to mutations inherited in an autosomal dominant manner located in one of three specific genes: *APP* (amyloid precursor protein), *PSEN1* (presenilin 1), and *PSEN2* (presenilin 2). The etiology of LOAD is considered multifactorial, with a strong genetic component, as heritability is estimated at 60–80% [30,35,36]. The strongest genetic risk factor for LOAD is the Apolipoprotein E (*APOE*) gene, namely, its *APOE* ε4 allele. Although *APOE* ε4 is the most significant known genetic factor associated with LOAD, it is neither necessary nor sufficient to cause the condition, and it is not the only genetic risk factor involved. Large-scale genome-wide association studies (GWASs) and whole-genome/exome sequencing have identified over 90 additional gene variants that influence the likelihood of developing the disease [37,38,39,40]. These variants are primarily linked to specific biochemical pathways—such as lipid metabolism, intracellular trafficking, inflammation, immune response, synaptic function, and transcription—underscoring their role in the development of LOAD.

The most important risk factor, aside from genetic background, is aging. Additionally, multiple environmental and lifestyle factors increase the risk of disease development. These include hypertension, type 2 diabetes, dyslipidemia, obesity, a low level of education, a lack of physical and mental activity, and others [41,42].

Currently, there is no proven approach to prevent or cure AD. The available therapeutic options (cholinesterase inhibitors and N-methyl-D-aspartate antagonists) are primarily symptomatic, offering only limited relief [43,44]. However, significant research and funding have been directed toward developing immunotherapeutic strategies targeting pathologic Aβ and tau proteins. Although preclinical animal studies have shown promising results, human clinical trials have yielded only modest success [45,46,47]. While recent anti-amyloid therapies approved by the United States Food and Drug Administration (FDA)—aducanumab, lecanemab, and donanemab—have successfully reduced Aβ plaques in the brain, only the latter two monoclonal antibodies have been shown to moderately slow cognitive decline [48]. However, this modest benefit comes with the risk of clinically significant harm [49]. Therefore, a substantial part of the research is dedicated to preventive strategies aimed at reducing the risk of developing the disease. Chronic inflammatory conditions, especially periodontitis, are of particular interest as multiple studies suggest that they may be one of the contributing factors in AD development.

## 3. Oral Microbiota and Periodontitis

The oral microbiota represents the second-most complex microbial community in the human body after the intestinal microbiota. Microorganisms inhabiting the oral cavity encompass around 700–1000 species with distinct subspecies [50,51,52]. Nevertheless, the average individual typically hosts around 100 to 200 species forming distinctive oral microbiomes [53,54,55]. Homeostasis is maintained through a delicate equilibrium between the resident microbiota and the host immune response. Disruption to this equilibrium, exacerbated by pathogenic bacterial species, is implicated in the development of inflammatory oral diseases, such as periodontitis [50,56]. The etiology is multifactorial, involving a combination of factors such as age, genetic background, diabetes mellitus, inadequate oral hygiene, and cigarette smoking [57].

Periodontitis is widely recognized as one of the most common chronic inflammatory conditions, characterized by the progressive destruction of the periodontium, including the periodontal ligament and alveolar bone, eventually leading to tooth loss [56]. While the disease can affect people of all ages, it is more commonly observed in the elderly. The estimated prevalence of periodontitis varies globally; however, it is generally considered quite high. Studies suggest that severe periodontitis affects approximately 10–11% of the global adult population [58,59]. When considering all stages of periodontitis, including mild and moderate forms, the prevalence can be as high as 20–50% in the global population and 70–90% in individuals aged over 65 years, depending on the population and diagnostic criteria used [51,60,61,62]. In the United States, for example, it is estimated that around 42% of adults aged over 30 years have some form of periodontitis [58]. As the population expands and ages, the numbers are anticipated to increase [63].

Periodontitis is treated through a combination of approaches depending on the severity of the condition, ranging from basic oral hygiene practices and professional mechanical debridement to antimicrobial therapy and periodontal surgery. Surgical interventions may include bone grafts, guided tissue regeneration, and growth factors, among other advanced techniques. Treatment primarily focuses on controlling the destruction of the tooth attachment apparatus caused by inflammation triggered by pathogens present in dental plaque [64]. However, periodontitis is not only a leading cause of tooth loss in adults. Over the past few decades, research into periodontitis and its associated pathogens has expanded beyond dentistry, with growing interest in their potential involvement in the onset and progression of various systemic diseases, including AD [8,25,65].

## 4. *Porphyromonas gingivalis*

The predominant etiological agents involved in the initiation and progression of human chronic periodontitis were traditionally considered to be members of the Red Complex triad: *Porphyromonas gingivalis*, *Tannerella forsythia*, and *Treponema denticola* [52,66,67]. Among these, *P. gingivalis* was identified as the key member of the subgingival microbiome, capable of remodeling the commensal bacterial community to promote a state of dysbiosis [8,68,69,70]. The interaction of *P. gingivalis* with other periodontal bacteria in the oral cavity supports the development and persistence of a biofilm, largely contributing to the overall pathogenicity of the microbial community by creating a protective environment that is resistant to host immune responses and antimicrobial treatments.

*P. gingivalis* is a Gram-negative, obligately anaerobic, oligotrophic rod that is classified in the family *Porphyromonadaceae*, order *Bacteroidales*, class Bacteroides, and phylum Bacteroidetes [71,72]. This bacterium is asaccharolytic and highly proteolytic, thus relying on the acquisition of peptides to ensure essential nutrients [72]. The genome of *P. gingivalis* consists of a single circular chromosome approximately 2.35 million base pairs in size, with coding sequences covering about 86%. Comparisons of the genomes of individual strains reveal high genetic diversity, especially in relation to their virulence factors.

*P. gingivalis* is mainly known as an opportunistic pathogen capable of efficiently colonizing the oral epithelium. Its specific niche is the subgingival sulcus, a shallow crevice between the tooth and gum that offers an ideal anaerobic environment. Within this protected area, *P. gingivalis* proliferates, inducing the dysbiosis of local commensals and the formation of biofilms [73]. As the bacterium multiplies, it secretes virulence factors, which trigger inflammation and degrade the surrounding periodontal tissues. This inflammatory response destroys the connective tissue and alveolar bone supporting the teeth, causing the gum tissue to detach from the tooth surface. As a result, the subgingival sulcus deepens, forming periodontal pockets that provide an even more favorable environment for *P. gingivalis* and other anaerobic bacteria species, allowing the biofilm to expand and further exacerbate the disease process [67]. According to Hajishengallis, *P. gingivalis* plays a coordinating role in inflammatory tissue damage and bone loss rather than being the direct cause, driving a cycle of inflammation that ultimately contributes to disease progression [74].

*P. gingivalis* is primarily linked to chronic periodontitis. The species has been found in 85.75% of subgingival plaque samples from patients with chronic periodontitis; however, it is also detectable at low levels in about 25% of individuals without oral disease [75,76]. In these individuals, *P. gingivalis* constitutes only up to 5% of the oral microbiota. As the disease develops, its representation increases [72]. The sustained presence of *P. gingivalis* in the periodontium relies on its capacity to escape the host immune system without suppressing the inflammatory response [77]. Throughout evolution, *P. gingivalis* has evolved distinct strategies based on manipulating immune mechanisms including intracellular signaling pathways, the complement system cascade, the cell cycle, apoptosis, and interactions with diverse host immune cell receptors [78].

## 5. Virulence Factors of *P. gingivalis* and Their Role in AD Pathogenesis

**Figure 1:**
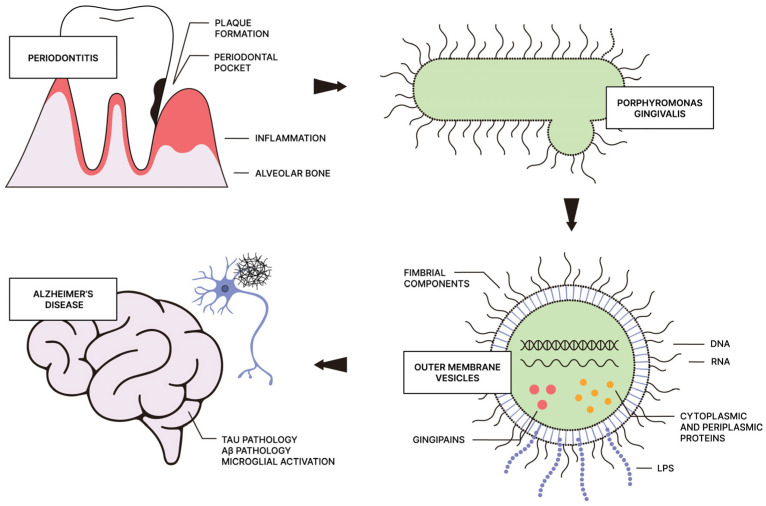
A schematic illustration showing *P. gingivalis* colonizing subgingival pockets in periodontitis and spreading its virulence factors via OMVs to the brain, potentially contributing to the development of Alzheimer’s pathology. Image created by the authors using Illustrator.

The pathogenicity and survival strategies of *P. gingivalis* depend on a myriad of virulence factors that enable the bacterium to colonize, invade, and persist in host cells [56,68]. Gram-negative bacteria, including *P. gingivalis*, generate outer membrane vesicles (OMVs) recognized as key virulence factors containing various other pathogenic components. Among the most significant are lipopolysaccharides (LPSs), fimbrial proteins, and proteolytic enzymes such as gingipains, described in detail in the following subsections. OMVs serve as efficient transmission vehicles, capable of releasing their contents into the extracellular environment or being internalized by host cells [79,80]. Following the invasion of periodontal tissues, *P. gingivalis* virulence factors are involved in the etiology of periodontitis by promoting dysbiotic inflammation and facilitating immune evasion [81]. However, their impact extends far beyond the periodontium and oral cavity, as they can disseminate to distant sites, underscoring the link between periodontal disease and systemic conditions, such as AD (Figure 1) [82,83].

*P. gingivalis* virulence factors promote brain inflammation and associated damage through diverse mechanisms (Table 1). Whether viable bacteria cells can cross the blood–brain barrier (BBB) and establish an infection within the brain remains debatable, especially given that the brain represents an unfavorable environment for anaerobic and asaccharolytic microorganisms like *P. gingivalis* [72]. In recent years, OMVs have gained attention as a likely mechanism for the distribution of the bacterium’s virulence factors, with the potential to reach the brain [20,72].

### 5.1. Outer Membrane Vesicles

OMVs are small, spherical nanostructures generated through the vesiculation of the outer membrane during all growth phases of the bacterium [157,158]. Vesiculation levels increase during bacterial stress, such as that which *P. gingivalis* experiences during the colonization of host tissues [159]. While some OMVs remain attached to the cell surface, significant numbers are released into the environment. Notably, OMVs are not byproducts of cell death, as they are generated without bacterial lysis and contain newly synthesized proteins [160]. OMVs can deliver a range of bioactive virulence-associated bacterial substances to host cells through several known mechanisms, primarily via endocytosis [79]. OMVs produced by colonizing bacteria like *P. gingivalis* can interact with and be internalized by different cell types, e.g., epithelial cells, dendritic cells, and macrophages, initiating a rapid immune response. The interaction of bacterial components with these cells is a well-known catalyst for triggering the inflammatory cascade [160].

OMVs range in size from 20 to 400 nm in diameter and consist of a proteo-liposomal bilayer membrane that encloses multiple cargo molecules [161]. The cargo of OMVs is diverse and contains mostly cell wall and periplasmic components trapped in the lumen during their release, e.g., LPS, peptidoglycan, and fimbrial proteins. OMVs can also be enriched with cytoplasmic proteins such as enzymes, heat shock proteins, iron capture proteins, and many others [20,80,161,162]. In this way, OMVs deliver molecular mediators involved in multiple biological processes such as interbacterial interactions, host–bacterium interactions, nutrient acquisition, biofilm formation, antimicrobial resistance, and immunomodulation [159,163]. Additionally, OMVs contain bacterial DNA and various types of RNA molecules, which can influence host gene expression following their internalization into host cells or can act as pathogen-associated molecular patterns (PAMPs) recognized by the host immune system [72,164,165].

*P. gingivalis* produces a significant number of OMVs that are considered highly virulent factors contributing substantially to the modulation and dysregulation of the host’s innate and adaptive immune responses [72,163,166]. Cecil et al. demonstrated that OMVs derived from *P. gingivalis*, *T. denticola*, and *T. forsythia* can trigger the secretion of proinflammatory cytokines from host immune cells, including tumor necrosis factor (TNF)-α, interleukin (IL)-1β, IL-8, IL-6, and macrophage inflammatory protein (MIP)-1α. They promote neutrophil recruitment and disrupt tight junctions in epithelial cells, facilitating the infiltration and activation of other immune cells, including monocytes and macrophages, thus contributing to the inflammatory response. OMVs can also induce the release of immunoregulatory cytokines, such as IL-10 [167].

A growing body of evidence suggests that OMVs play a pivotal role in translocating *P. gingivalis* virulence factors from the periphery into the central nervous system (CNS), thereby promoting AD pathologies. Animal-based studies and in vitro BBB models have collectively demonstrated that circulating OMVs containing proteases can breach the BBB by invading cerebral microvascular endothelial cells and degrading or downregulating tight junction proteins, such as zonula occludens (ZO-1), claudin-5, and occludin [84,85,86]. This disruption increases BBB permeability, allowing inflammatory mediators, leukocytes, bacterial virulence factors, and possibly *P. gingivalis* to enter the brain through the paracellular pathway. In addition to blood pathways, OMVs and virulence factors may also translocate into the CNS via the trigeminal nerve [87]. Upon reaching regions such as the hippocampus, cortex, cerebral ventricles, or choroid plexus, OMVs impair memory and learning ability, promote tau phosphorylation in neurons, activate astrocytes and microglia, and promote neuroinflammation [86,87,88] by delivering *P. gingivalis* virulence factors and triggering nuclear factor kappa-light-chain-enhancer of activated B cells (NF-κB) signaling [87,88], the NLR family pyrin domain-containing 3 (NLRP3) inflammasome [86], or toll-like receptor (TLR)2-dependent, cathepsin (Cat)B/CatL-independent phagocytic mechanisms [89]. When endocytosed by neurons, OMVs induce neurotoxicity and severe AD-like degradation [83,87,90], likely by suppressing N-methyl-D-aspartate receptor (NMDAR)-dependent neuronal brain-derived neurotrophic factor (BDNF) signaling [87].

Recently, another mechanism for delivering *P. gingivalis* virulence factors to the CNS has been proposed: the cellular secretion of small extracellular vesicles or exosomes. These vesicles, originating from *P. gingivalis*-infected cells, contain gingipains and other virulence factors. They have been shown to penetrate the brains of recipient experimental mice after oral injection. Exosomes obtained from human donors with periodontitis have induced IL-1β and IL-6 production, increased BBB permeability, and crossed the BBB in vitro. These findings suggest that microbial-induced exosomes can disseminate from the oral cavity and act as vehicles transporting *P. gingivalis* virulence factors to the brain [168,169].

### 5.2. Fimbriae

The initial steps of infection, i.e., the attachment of *P. gingivalis* to the oral tissue and the invasion of the host cells, are mediated by fimbriae (or pili)—slender, protein-based surface appendages extending from the outer membrane of the bacterial cell. Two main distinct types of fimbriae have been identified: long (major) fimbriae encoded by the FimA gene and short (minor) fimbriae encoded by the Mfa1 gene [76,170]. Accessory fimbriae are encoded by downstream genes fimB, fimC, fimD, and fimE and play a role in binding to matrix proteins and interacting with the CXC chemokine receptor type 4 (CXCR4) [105]. Fimbriae enable the bacterium to bind to diverse host cells, especially epithelial cells, macrophages, and dendritic cells. They show substantial binding affinity to various host cell membrane molecules, such as integrins and ICAM-1, and extracellular matrix proteins and glycoproteins, such as vitronectin, fibrinogen, and fibronectin, which play vital roles in cellular signal transduction [91]. Due to their adhesive functions, fimbriae promote the co-aggregation of *P. gingivalis* with other bacteria species, contributing to the formation and stability of polymicrobial dental biofilms [56,171]. Different *P. gingivalis* strains exhibit variations in fimbriae morphology and their ability to adhere to host cells [172]. Although fimbriae have traditionally been recognized for the mentioned strong adhesive properties, recent research suggests their involvement in subverting the host immune system and sustaining chronic inflammation.

Major fimbriae (FimA) have been shown to promote inflammatory responses by inducing TLR2-dependent adhesion, transendothelial migration, and the activation of monocytes and macrophages, resulting in the robust secretion of predominantly proinflammatory cytokines, including IL-1β, IL-6, IL-8, TNF-α, and GM-CSF [92,93,94,95,96]. The FimA-induced TLR2-mediated inflammatory response exhibits a strong dependence on binding to the membrane CD14 molecule and on cooperation with TLR1 or TLR6 [94]. Additionally, FimA facilitate the invasion of endothelial cells by *P. gingivalis* and induce the expression of chemokines (e.g., MCP-1 and IL-8) and adhesion molecules (e.g., ICAM-1, VCAM-1, P-selectin, and E-selectin) by endothelial cells, thereby promoting the transmigration of inflammatory cells [100,101,102]. Furthermore, Mfa1 and accessory fimbrial subunits (FimC, FimD, and FimE) modified by *P. gingivalis* peptidyl arginine deiminase (PPAD) contribute to the proinflammatory host cell response to bacteria by activating TLR2 [97,98,99].

Concurrently, *P. gingivalis* fimbriae can manipulate host immune mechanisms, facilitating immune evasion and pathogen survival. FimA interact with complement receptor 3 (CR3; CD11b/CD18) on monocytes and macrophages, leading to extracellular signal-regulated kinase 1/2 (ERK1/2) phosphorylation and the suppression of LPS-induced IL-12 production [93,103]. Moreover, FimA induce the TLR2-dependent production of IL-10 in T cells and CD11b+ myeloid cells, which suppresses the initial IFN-γ T-cell response to *P. gingivalis*, thereby aiding the pathogen in evading host immunity [107]. Another strategy involves the simultaneous engagement of TLR2 by FimA and chemokine receptor CXCR4 by FimC, FimD, and FimE. This interaction induces TLR2-CXCR4 crosstalk, which inhibits the MyD88-dependent antimicrobial pathway and undermines the nitric oxide (NO)-dependent killing function of monocytes and macrophages. Additionally, fimbrial binding to CXCR4 induces CR3 activation in a TLR2-independent manner, further impairing *P. gingivalis* clearance and enabling its safe entry and survival within macrophages [104,105,106].

Minor fimbriae Mfa1 also contribute to immune subversion by interacting with the DC-SIGN receptor on dendritic cells (DCs). This interaction activates the Akt/mTOR signaling pathway, which allows *P. gingivalis* to evade autophagic destruction within DCs. Furthermore, it influences DC maturation, apoptosis, cytokine production, and antigen presentation, facilitating pathogen survival, immune evasion, and dissemination [108,109,110,111,112]. Both FimA and Mfa1 also induce the differentiation of monocytes into myeloid-derived dendritic suppressor cells, which inhibit cytotoxic T lymphocytes and induce FoxP3+ T regulatory cells, thereby promoting immunosuppression [113]. The ability of *P. gingivalis* to simultaneously drive dysbiotic chronic inflammation and evade immune clearance creates a favorable environment for its dissemination and the spread of its virulence factors, ultimately contributing to neuroinflammation associated with AD.

### 5.3. Lipopolysaccharides

The major structural virulence factors of *P. gingivalis* include lipopolysaccharides (LPSs), which are integral components of the outer membrane of Gram-negative bacteria. LPSs are large glycoconjugates composed of a hydrophobic lipid domain (lipid A) attached to a core oligosaccharide and a distal polysaccharide referred to as the O antigen [56,76]. As PAMPs, LPSs serve as potent activators of the host innate immune response by engaging pattern recognition receptors (PRRs), primarily TLR2 and TLR4. The binding properties and activation of particular TLRs by LPSs depend mainly on the chemical structure of lipid A. Atypically, *P. gingivalis* LPSs display structural heterogeneity in their lipid A domain, enabling the bacterium to modulate its interactions with host immune receptors and evade immune responses or induce inflammation selectively [56,173]. More specifically, lipid A can exist in two dominant forms, tetra-acylated and penta-acylated, each influencing its interaction with TLRs expressed on the host immune cells differently. The stimulatory activity of the tetra-acylated form seems to rely predominantly on TLR2, while its interaction with TLR4 may even be antagonistic, inhibiting classical TLR4 signaling pathways associated with strong proinflammatory responses. This mechanism enables *P. gingivalis* to evade robust immune responses and hinder effective immune clearance. In contrast, the penta-acylated form may activate both TLR2 and TLR4, triggering downstream signaling pathways associated with the production of proinflammatory cytokines such as IL-1β, TNF-α, IL-6, IL-8, and CCL2 [56,61,173,174]. Interestingly, a recent study on *P. gingivalis* LPS-stimulated microglial cells reported the upregulation of proinflammatory factors, including IL-1β, IL-6, TNF-α, IL-17, and IL-23, via the activation of the TLR2/4-mediated NF-κB/STAT3 signaling pathways. However, this effect was less robust than stimulation with Escherichia coli LPSs [136], potentially facilitating chronic, low-grade inflammation. The possession of multiple diverse lipid A moieties also makes the bacterium more difficult to detect by the host’s innate immune mechanisms, thereby supporting the maintenance of its virulence [175,176].

An increasing number of studies have identified links between *P. gingivalis* LPSs and AD pathogenesis (Table 1). Both *P. gingivalis* and its LPSs have been detected in the post-mortem brain tissues of patients with AD, suggesting that the bacterium can reach the brain during their lifetime [26]. Additionally, LPSs may enter the CNS as cargo within OMVs or through phagocytosis by peripheral immune cells, which then adhere to endothelial cells of the BBB and penetrate the brain. Endothelial cells may also facilitate LPS transport by binding to the pattern recognition receptor CD14/TLR4 complex. Furthermore, free LPSs can cross the BBB disrupted by proinflammatory cytokines or form complexes with lipopolysaccharide-binding proteins (LBPs), which are subsequently transported into the brain via specific endothelial receptors [177]. Emerging evidence from in vitro and animal experiments, using either wild-type rodents or mouse AD models exposed to *P. gingivalis* or its LPSs, indicates that upon entry into the CNS, LPSs induce or exacerbate cognitive impairment and AD-like pathologies, including microglia-mediated neuroinflammation, gliosis, Aβ accumulation, the formation of hyperphosphorylated tau and NFTs, and neurodegeneration [23,24,25,83,89,114,115,116,117,118,119,120,121,122,123,124,125,126,127,128,129,130,131,132,133,134,135,136,137,138,139,140,141,142,178,179,180,181,182,183,184,185,186,187]. LPSs may employ diverse strategies and pathomechanisms to contribute to these outcomes. Within the CNS, LPSs disrupt Aβ homeostasis by both increasing Aβ production and reducing its degradation [23]. The former is driven by the upregulation of amyloid precursor protein (APP) and APP-cleaving enzymes, including β-secretase (BACE1) and γ-secretase subunits presenilin 1 (PS1) and presenilin 2 (PS2) [116,122,125], while the latter is thought to result from the decreased expression of Aβ-degrading enzymes, such as neprilysin [123]. Recent studies also propose that a significant proportion of *P. gingivalis*-related cerebral amyloid may originate from the periphery, as chronic *P. gingivalis* infection has been shown to expand circulating Aβ pools in the plasma and peripheral inflammatory monocytes and macrophages [115,185,188]. The subsequent influx of Aβ from the periphery into the brain is likely facilitated by the CatB/NF-κB-mediated upregulation of the receptor for advanced glycation end products (RAGE) in cerebral endothelial cells [118].

LPSs derived from *P. gingivalis* significantly contribute to the development of tau pathology by increasing tau expression and promoting its hyperphosphorylation [24,28,125,126,142]. Particularly, LPSs increase the activity of glycogen synthase kinase-3β (GSK-3β) in both neurons and microglia, suggesting that GSK-3β is involved in tau hyperphosphorylation and the subsequent development of learning and memory deficits [126]. In addition, LPSs indirectly induce tau hyperphosphorylation by triggering the synthesis and release of the proinflammatory cytokine IL-1β, which has been shown to attenuate the activity of protein phosphatase 2A (PP2A) in neurons [127]. The LPS-induced decrease in PP2A activity through phosphorylation may also be mediated by the increased expression of cofilin 2 [128], a protein central to the regulation of actin filament dynamics and previously proposed as a biomarker for AD [189]. Moreover, LPSs also promote neurotoxicity and neurodegeneration by exacerbating the production of reactive oxygen species and inducing oxidative stress, impairing insulin sensitivity, and causing mitochondrial dysfunction [120,138,141,142,180].

*P. gingivalis* LPSs have also been shown to induce the synthesis of several proinflammatory cytokines, including IL-1β, IL-6, TNF-α, IL-8, IL-17, and IL-23, by microglial cells and drive their polarization to the M1 phenotype via the TLR2/4-mediated NF-κB signaling pathway, thereby contributing to microglia-driven neuroinflammation and neurodegeneration [23,125,126,131,132,136,138]. Interestingly, the LPS-induced synthesis and processing of the key proinflammatory and neurotoxicity-promoting cytokine IL-1β appears to depend on CatB [114]. LPSs significantly increase the expression of CatB [114], while the phagocytosis of *P. gingivalis* or Aβ can induce the leakage of CatB from destabilized lysosomes into the cytoplasm [135,190,191]. CatB upregulates pro-IL-1β and NLRP3 in an NF-κB-dependent manner [192] and facilitates the processing of pro-IL-1β into mature IL-1β through the direct or NLRP3-dependent activation of pro-caspase-1 [89,193]. The antimicrobial peptide human β-defensin 3 (hBD3) has been shown to inhibit CatB (and CatL) activities, thereby suppressing the oxidative and inflammatory responses of microglia [89,140]. Thus, the hBD3-mediated inhibition of CatB could potentially offer a therapeutic approach to mitigate the IL-1β-associated inflammatory cycle.

In chronic periodontitis, LPSs derived from *P. gingivalis* stimulate peripheral monocytes and macrophages to produce proinflammatory mediators such as TNF-α and IL-1β, which can compromise the integrity of the BBB and, upon penetrating the brain, exacerbate neuroinflammation. Interestingly, leptomeningeal cells have also been shown to transduce these LPS-induced inflammatory signals from peripheral macrophages to brain-resident microglia, promoting their polarization into a proinflammatory M1-like phenotype during chronic periodontitis. Moreover, leptomeningeal cells appear to contribute to synaptic dysfunction through CatB/NLRP3 inflammasome-mediated IL-1β production following *P. gingivalis* phagocytosis [132,135]. Another mechanism by which *P. gingivalis* and chronic periodontitis may induce or exacerbate AD involves gut–brain axis crosstalk [179]. Animal studies employing APP/PS1 transgenic mice have suggested that ingested periodontitis-related microbiota, such as *P. gingivalis* and its LPSs, could exacerbate AD progression by disrupting intestinal homeostasis and inducing gut microbial dysbiosis, intestinal inflammation, and intestinal barrier impairment. This sequence of events eventually leads to peripheral inflammation, BBB disruption, neuroinflammation, and synaptic impairment [124,183].

### 5.4. Gingipains

Among powerful virulence factors are enzymes known as gingipains, which function as both cell surface and secretory cysteine proteases. They are responsible for at least 85% of the overall proteolytic activity that *P. gingivalis* exerts [61,77,194,195]. Based on their substrate specificity, gingipains encompass arginine-specific gingipains A (RgpA) and B (RgpB) which cleave peptide bonds after arginine residues, and the lysine-specific gingipain (Kgp) which disrupts peptide bonds after lysine residues [143]. These enzymes are essential for the survival of *P. gingivalis* as they play crucial roles in many pathways. They facilitate host colonization and tissue degradation [143,145]. The bacterium uses gingipains for nutrient acquisition as they break down host proteins into smaller peptides and amino acids, which serve as nutrient sources. In addition to the action on host substrates, they also act in the post-translational processing of *P. gingivalis* proteins. Gingipains can directly participate in co-aggregation with other periodontal bacteria, thereby enhancing the development and stability of complex polymicrobial biofilms [81]. They also contribute to the dysregulation of key immune mechanisms by directly degrading cell surface receptors, immunoglobulins, cytokines, chemokines, and complement components [56,76,77,143,144,145]. *P. gingivalis* evades complement-mediated lysis by using gingipains to degrade complement components C3 and C5, thereby inhibiting the deposition of C3b on the bacterial cell surface [176]. These mechanisms provide protection to bacteria, facilitate immune evasion, and promote dysbiotic inflammation.

Gingipains are potent agents, capable of influencing AD pathology through various mechanisms. They can be displayed on the surface of OMVs, which allows them to interact directly with host tissues and cells. They can also be contained within the OMVs and protected from the extracellular environment until they are released at the target site [80]. The effects of gingipains contribute to the disruption of the BBB through the degradation of tight junction proteins [84,85], thereby facilitating the entry of *P. gingivalis* and its virulence factors into the brain parenchyma. Another mechanism that may increase BBB permeability involves the major facilitator superfamily domain-containing 2a (Mfsd2a)/Caveolin-1 (Cav-1)-mediated transcytosis pathway. In this process, gingipains upregulate Cav-1 and promote caveolae formation in brain microvascular endothelial cells (BMECs) by inhibiting Mfsd2a expression, which, in turn, promotes transcytosis [146,147]. A compromised BBB allows the entry of immune cells, inflammatory mediators, and potentially neurotoxic substances into the brain. This is one of the mechanisms by which gingipains contribute to inflammation and neuronal damage, promoting neurodegenerative processes. Gingipains also assist in inducing inflammatory responses in the brain by activating immune cells and stimulating the release of proinflammatory cytokines and chemokines. Arginine- and lysine-specific gingipains cooperatively contribute to the *P. gingivalis*-induced cell migration and expression of proinflammatory mediators (IL-6, TNF-α, and iNOS) by microglia through the activation of protease-activated receptor 2 (PAR2) and the subsequent activation of the phosphoinositide 3-kinase (PI3K)/Akt and mitogen-activated protein kinase (MAPK)/ERK kinase (MEK)/ERK pathways [148,149].

Several human and animal studies as well as in vitro models have pointed to the translocation of gingipains into the CNS and their potential role as drivers of AD pathology. A pivotal study by Dominy et al. provided evidence of *P. gingivalis* Kgp and Rgp antigens in the brain autopsy specimens of individuals with AD [25]. Gingipains were also detected in control human brain specimens but in lower amounts than in AD brains. Their loads correlated with tau and ubiquitin pathology as well as with the severity of neurodegeneration [25]. Remarkably, oral *P. gingivalis* administration in mice caused the increased production of Aβ42, a major component of amyloid plaques and a typical hallmark of AD. Neurotoxic effects of gingipain proteases on tau protein were observed both in vivo and in vitro. They were supposed to be caused by the cleavage of tau at specific sites, disrupting its normal function and potentially contributing to the formation or propagation of tau tangles inside neurons [25]. In a study with ApoE^−/−^ mice, chronic gingival infection with *P. gingivalis* showed the intracerebral dissemination of the pathogen and the presence of gingipains in microvessels of the hippocampus, along with signs of physical tissue damage in the brains of infected mice [22]. Furthermore, the repeated exposure of wild-type mice to orally administered *P. gingivalis* resulted in neuroinflammation, neurodegeneration, microgliosis, astrogliosis, and the formation of phosphorylated tau, intracellular NFTs, and extracellular amyloid plaques. Gingipains could be detected in the hippocampi and were localized extracellularly, intranuclearly, and perinuclearly in microglia, astrocytes, and neurons [24]. In mice injected with *P. gingivalis* OMVs into the abdominal cavity, gingipains were detected in the region around cerebral ventricles, the choroid plexus, and ventricular ependymal cells and promoted tau phosphorylation with neuroinflammation [88]. In vitro, *P. gingivalis* was shown to infect neurons and produce proteolytically active gingipains [28]. The resulting neurotoxicity and AD-like pathology included the accumulation of autophagic vacuoles and multivesicular bodies, cytoskeleton disruption, the degradation and phosphorylation of tau, and synapse loss [28,83,88].

The pathogenic effects of gingipains can be attributed to several mechanisms. In addition to disrupting the integrity of the BBB and facilitating inflammation-mediated tissue injury, gingipains may also directly contribute to Aβ and tau pathology, ultimately leading to neuronal dysfunction and synaptotoxicity. This could be achieved through the activation of caspase-3 [150], an enzyme that promotes APP cleavage [151], tau cleavage [152,153], and tau phosphorylation [154]. The latter effect is mediated by the GSK-3β kinase pathway via the caspase-3-dependent cleavage of protein kinase B (Akt). Cleavage produces a smaller 45 kDa fragment of Akt and decreases the formation of two phosphorylated active isoforms, namely, p-Akt (Thr308) and p-Akt (Ser473). The lower levels of active Akt lead to the reduced phosphorylation of the principal tau kinase GSK-3β, resulting in its increased activity and, consequently, in greater tau phosphorylation [154]. Both gingipains and LPSs also cause a marked downregulation of G protein-coupled receptor kinase 5 (GRK5) [130]. Its deficiency is considered a causal risk factor for AD [196]. The effect of gingipains on GRK5 expression in neurons is likely mediated by PARs, leading to the increased expression of the pro-apoptotic signaling molecule p53 and elevated levels of phosphorylated tau [130]. The p53 molecule has been shown to interact with GSK-3β [197], promoting tau phosphorylation and cell death, while GRK5 may act as a regulator of this p53/GSK-3β axis [130,198,199]. Additionally, GRK5 downregulation may enhance NF-kB activity, leading to increased iNOS-induced oxidative damage, production of proinflammatory cytokines [130,199], and Aβ accumulation [185]. Thus, the downregulation of GRK5 by LPSs and gingipains likely represents a major mechanism underlying *P. gingivalis*-induced neurodegeneration.

The RgpB gingipain has also been identified as a potent activator of the metalloproteinase meprin β [200], which acts as an alternative β-secretase of APP, capable of increasing brain Aβ levels and promoting learning and memory impairments [201,202]. In vitro, an RgpB-mediated increase in meprin β activity has led to massive generation of Aβ-peptides, one of the hallmarks of AD [155]. Gingipains have also been shown to cleave apolipoproteins, including ApoE. In the brain, ApoE plays a crucial role in mediating cholesterol transport to neurons and facilitating the breakdown of Aβ peptides. The fragmentation of ApoE by gingipains may impair its function, potentially hindering Aβ clearance from the brain. This dysfunction could contribute to a localized atherogenic process and promote Aβ accumulation in the brain [156].

To counter the effects of gingipains on AD pathology, Dominy et al. developed small-molecule inhibitors targeting gingipains, which were reported to successfully reduce the bacterial load in the brain, block Aβ42 production, decrease neuroinflammation, and protect neurons in the hippocampus [25]. These findings strongly suggest that gingipain inhibitors could be a promising therapeutic approach for addressing *P. gingivalis* brain colonization and subsequent neurodegeneration in AD [203]. Undoubtedly, the Dominy et al. study has sparked significant interest in the potential role of *P. gingivalis* in AD, highlighting the need for further research.

### 5.5. Other Virulence Factors

While OMVs, fimbriae, LPSs, and gingipains are the most extensively studied virulence factors implicated in AD pathogenesis, other components of *P. gingivalis* may also contribute through diverse mechanisms. For instance, phosphoglycerol dihydroceramide (PGDHC), a sphingolipid of *P. gingivalis*, has been shown to upregulate the secretion of soluble Aβ42 peptide and the expression of APP, induce the hyperphosphorylation of tau protein, and promote neuronal senescence, as indicated by the production of senescence-associated secretory phenotype (SASP) markers [204]. In another study, capsular polysaccharides appeared to play a central role in the development of AD-like pathology triggered by *P. gingivalis*, as only encapsulated strains were found to promote neuroinflammation, changes in astrocytic morphology, and Aβ and tau pathology in the hippocampus, ultimately leading to memory deficits [205]. Bacterial molecular chaperones, such as heat shock protein (HSP) family members GroEL/GroES (homologs of eukaryotic Hsp60/Hsp10), DnaK/DnaJ/GrpE (Hsp70/Hsp40/Hsp70 cofactor), HtpG (Hsp90), and Clp (Hsp100) and other stress-related proteins, are also putative contributors. These chaperones play a pivotal role in maintaining bacterial proteostasis and enabling adaptation to stressful conditions, including heat shock, oxidative stress, nutrient deprivation, and host defense mechanisms [206,207,208]. It can be hypothesized that *P. gingivalis* chaperones contribute to chronic periodontitis and AD pathology by aiding in the proper folding, stabilization, and functional maintenance of key virulence factors like gingipains and fimbriae [206], promoting host cell invasion [207,209], triggering inflammatory responses [210,211,212,213], or impacting endothelial cell functions [214,215,216]. While human HSP chaperones play complex and often opposing roles in Aβ and tau pathology as reviewed in [217,218,219,220,221], the involvement of *P. gingivalis* HSPs in these processes remains an open question. For instance, bacterial DnaK, together with its cochaperones DnaJ, GrpE, and ClpB, has been shown to dissolve large, amorphous aggregates and disassemble tau fibril superstructures into single fibrils [222]. However, unlike its human counterpart, the bacterial Hsp70 chaperone system seems to lack the ability to disaggregate single tau fibrils into monomeric and small oligomeric species with seeding and toxic properties [219,222]. Hence, it remains to be determined whether the activities of *P. gingivalis* HSPs directly contribute to the development of AD pathologies.

## 6. Concluding Remarks on the Association Between AD and *P. gingivalis*

Unraveling the causative mechanisms behind late-onset AD remains a significant challenge. While the complex etiopathogenesis of this neurodegenerative disorder is not fully understood, it is clear that it arises from multiple contributing factors. As reviewed in this paper, compelling evidence suggests a potential multifaceted role of *P. gingivalis* in the development and progression of AD. The exact mechanisms by which the bacterium influences disease development are not yet sufficiently elucidated; however, *P. gingivalis* may contribute to AD pathogenesis through direct and indirect processes. The initially postulated direct pathway supposes the ability of *P. gingivalis* to reach and infect the brain either via cranial nerves or hematogenous spread. *P. gingivalis* residing in the periodontium may enter the bloodstream during dental treatments such as brushing via vascular channels in the gingival sulcus area, which has a high microbial load in individuals with periodontitis. Subsequently, bacteria disseminate through systemic circulation and reach the brain by crossing the BBB, perivascular spaces, or circumventricular organs [176,223]. However, the presence of *P. gingivalis* as an actively infecting or colonizing agent in human brains remains uncertain. Although studies have identified *P. gingivalis* DNA, gingipains, and other virulence factors in brain tissue from patients with AD, these findings do not conclusively demonstrate a vital, actively colonizing infection. Detecting live *P. gingivalis* in brain tissue would require evidence from techniques such as the culturing of bacteria from brain samples, which is challenging due to the anaerobic conditions the bacterium requires and the technical limitations in processing human brain tissue. Therefore, while *P. gingivalis* components are indeed found in the brain, confirming active infection remains an area for further research. The latest studies suggest that more probably than the entire bacteria, OMVs released by *P. gingivalis* primarily deliver virulence factors to the brain tissues [72]. Gingipains mediated by OMVs have been shown to disrupt tight junctions in cerebral endothelial cells, increasing BBB permeability and allowing the entry of other virulence factors and inflammatory mediators into the brain, potentially contributing to neuroinflammation, neuronal damage, and brain atrophy [72,85]. OMVs of *P. gingivalis* are still significant research subjects for understanding periodontal disease and exploring their potential connections to systemic conditions such as AD. Multiple pieces of evidence also support the indirect hypothesis pointing out the role of a proinflammatory state in chronic periodontal infections. Chronic periodontitis induces low-grade systemic inflammation with the overexpression of peripheral inflammatory mediators that are supposed to promote neuroinflammatory events in the brain and exacerbate AD pathology [56,143,223,224,225,226].

The authors recognize that this review is limited by its focus on the relationship between a single pathogen and AD. However, the matter may be far more complex, as microbiota-related diseases are often driven by the collective actions of multiple microbial species. Numerous microorganisms are suspected to be involved in AD pathology, including other oral and non-oral bacteria species (e.g., *Borrelia burgdorferi*, *Chlamydia pneumonia*), viruses (herpes simplex type I), and fungal genera (*Alternaria*, *Botrytis*, *Candida*, and *Malassezia*) [53,227,228,229,230,231,232]. Recent evidence indicates that periodontal pathogens including *P. gingivalis* can alter the composition of the intestinal microbiome both directly and indirectly [233]. The “microbiota–gut–brain axis” connects gut dysbiosis to immune activation via a compromised gut barrier, triggering a systemic inflammatory response. This inflammation facilitates the disruption of the BBB, promoting neuroinflammation and ultimately contributing to neural degeneration, as observed in AD [124,179,183,234,235,236]. Investigating and understanding the intricate interactions among these organisms poses a significant challenge. Ultimately, besides the impact of the host immune system and the microbiome, complex diseases like AD arise from the dynamic interplay between genetic background, pre-existing comorbidities, and various demographic, social, and lifestyle factors.

Finally, it is important to note that the association between periodontitis and AD is likely bidirectional, with each condition potentially exacerbating the other. On the one hand, numerous studies indicate that chronic periodontitis may contribute to the development of AD. On the other hand, cognitive impairments commonly seen in patients with AD can lead to difficulties with oral motor skills (e.g., swallowing, brushing) and challenges in maintaining proper oral hygiene. These issues increase the risk of developing or worsening periodontitis, which, in turn, may further support AD pathology [44,65,237].

Currently, there is no effective treatment for AD, nor is there a single theory that fully explains its etiopathogenesis. Understanding the molecular mechanisms underlying the observed connections between periodontitis and AD is crucial for elucidating the etiology of late-onset AD, which is essential for developing effective intervention and treatment strategies. The management of infectious oral diseases, particularly periodontitis, which reduces the anaerobic microbial load, may also contribute to preventing neurodegenerative disorders such as Alzheimer’s.

## Figures and Tables

**Table 1:** The effects of *P. gingivalis* virulence factors on the immune system and the pathogenesis of Alzheimer’s disease.

Virulence Factor	Effects	References
OMVs	Impair BBB integrity and increase its permeability by downregulating or disrupting tight junction proteins, likely by delivering gingipains into the cerebral microvascular endothelial cells.	[84,85,86]
	Promote the translocation of *P. gingivalis* virulence factors into the brain through the trigeminal nerve.	[87]
	Impair memory and learning ability, promote tau phosphorylation in neurons, activate both astrocytes and microglia, and promote neuroinflammation.	[86,87,88,89]
	Induce neurotoxicity and severe AD-like degradation when endocytosed by neurons.	[83,87,90]
Fimbriae	Promote bacterial motility, auto- and co-aggregation with other bacteria, biofilm formation, adhesion to various host cell membrane molecules and extracellular matrix proteins, and the invasion of host cells.	[61,91]
	FimA induce adhesion, transendothelial migration and activation of monocytes and macrophages, and the secretion of predominantly proinflammatory cytokines.	[92,93,94,95,96]
	Mfa1 and accessory fimbrial subunits (FimC, FimD, and FimE) promote proinflammatory host cell response.	[97,98,99]
	Mediate *P. gingivalis* invasion of endothelial cells and induce the expression of chemokines and adhesion molecules by endothelial cells.	[100,101,102]
	FimA promote the immune evasion of *P. gingivalis* by inhibiting IL-12 production and IFN-γ-mediated T-cell response, while FimC, FimD, and FimE suppress macrophage-mediated NO-dependent killing mechanisms.	[93,103,104,105,106,107]
	Mfa-1 promotes survival and immune evasion by influencing DC functions and helping *P. gingivalis* to subvert autophagic destruction within DCs.	[108,109,110,111,112]
	FimA and Mfa1 promote immunosuppression and immune evasion by inducing the differentiation of monocytes to MDDSCs, which inhibit CTLs and induce FoxP3+ Tregs.	[113]
LPSs	Increase BBB permeability and promote its disruption by inducing the secretion of proinflammatory cytokines.	[84]
	Promote Aβ accumulation and pathology by increasing the production of Aβ, enhancing the activity of APP-cleaving enzymes, decreasing Aβ degradation, and increasing the peripheral pool of Aβ.	[23,114,115,116,117,118,119,120,121,122,123,124,125]
	Promote tau pathology by facilitating tau phosphorylation, aggregation, and NFT formation.	[120,125,126,127,128,129,130,131]
	Promote neuroinflammation by activating microglial cells and stimulating the production of proinflammatory cytokines (e.g., IL-1β, TNF-α, IL-6) and reactive oxygen species.	[23,89,114,116,117,119,120,122,124,125,126,131,132,133,134,135,136,137,138,139,140]
	Induce oxidative stress, mitochondrial dysfunction, neurotoxicity, and synaptic loss, thereby contributing to neurodegeneration.	[83,130,131,138,141,142]
Gingipains	Promote microbial dysbiosis and immune evasion by degrading cell surface receptors, immunoglobulins, cytokines, chemokines, and complement components.	[56,76,77,143,144,145]
	Increase BBB permeability by degrading tight junction proteins and promoting transcytosis.	[84,85,146,147]
	Contribute to the *P. gingivalis*-induced cell migration and expression of proinflammatory mediators (IL-6, TNF-α, and iNOS) by microglia, resulting in microgliosis and neuroinflammation.	[88,148,149]
	Promote APP cleavage and Aβ accumulation, facilitate tau cleavage and phosphorylation, and induce neurotoxicity and AD-like pathology.	[25,28,83,88,130,150,151,152,153,154,155,156]

Aβ: amyloid beta; AD: Alzheimer’s disease; APP: amyloid beta precursor protein; BBB: blood–brain barrier; CTL: cytotoxic T cell; IFN-γ: interferon gamma; IL: interleukin; iNOS: inducible nitric oxide synthase; LPSs: lipopolysaccharides; MDDSC: myeloid-derived dendritic suppressor cell; NFT: neurofibrillary tangle; NO: nitric oxide; OMVs: outer membrane vesicles; TNF-α: tumor necrosis factor alpha; Treg: T regulatory cell.

## Data Availability

No new data were created or analyzed in this study.
